# Prevalence of Violence against Children in Families in Tripura and Its Relationship with Socio-economic Factors

**DOI:** 10.5249/jivr.v2i1.31

**Published:** 2010-01

**Authors:** Sibnath Deb, Subhasis Modak

**Affiliations:** ^*a*^Visiting Faculty, School of Public Health, Faculty of Health Queensland University of Technology Brisbane, Australia.; ^*b*^Post-doctoral Fellow, Department of Applied Psychology Calcutta University, Kolkata, India.

## Abstract

**Background:**

Violence against children is a deep-rooted social problem in India. The problem is also related to economic as well as cultural beliefs and practices. The objective of this study was to ascertain the prevalence and nature of violence experienced by the children in families in Tripura, India and its relationship with socio-economic factors.

**Methods:**

A group of 320 children (160 males and 160 females) studying in Class VIII and IX and aged between 14-19 participated in the study after obtaining their informed consent from eight randomly selected English and Bengali medium schools in Agartala, Tripura (India). Data were collected by using a specially designed 'Semi-structured Questionnaire.

**Results:**

Findings revealed that about 20.9% (67/320), 21.9% (70/230) and 18.1% (58/230) of the children experienced psychological, physical and sexual violence respectively. Male children were more likely to be victims of psychological and physical violence while female children experienced more sexual violence (p less than 0.01).Further analysis of data revealed some relationship between violence against children and nuclear family(p was less than 0.01),uncongenial and/or disturbed family environment (p was less than 0.01)and dominating, short-tempered and/or aggressive parent personality (p was less than 0.01),irrespective of the nature of the violence. Physical violence was found to be more prevalent in high income families (p was less than 0.01) while children from the lower income group of families experienced more psychological violence (p was less than 0.01). Sexual violence was found to be equally prevalent in all socio-economic groups. The study also clearly indicated that academic performance of violence-experienced children, irrespective of nature of violence and socio-economic groups was poor compared to academic performance of non-violence-experienced children (p was less than 0.01).

**Conclusions:**

About one-fifth of the children under study did experience violence in Tripura. Findings speak in favor of an intervention program for creating awareness among parents and teachers about the issue of violence against children, targeted at parents when they meet for periodic parent-teachers meetings in the educational institutions.

## Introduction

Violence against children in any form constitutes a violation of the basic rights of children. During childhood, children deserve unconditional love and affection from their parents and basic minimal facilities for proper physical, social, mental and career development. Therefore, it is important to discipline children in a friendly manner instead of applying any force and/or threat. It is the responsibility of the family members and/or guardians to protect children from all forms of violence and guide them to become responsible and productive citizens of society. Family is a place to be considered as warm, safe, personal, and peaceful.^1^ A basic assumption of the United Nation Convention on the Rights of The Child (CRC) is that the family is the natural environment for the growth and well-being of all its members – particularly for children.^2^

Unfortunately, the family is also the most common place where children experience different forms of violence owing to a number of causal and contributory factors such as stress, poverty, living conditions, marital discord, psychiatric and/or psychological problems of the adults at home, poor enforcement of law, lack of child protection policies and so on.^3-8^ The prevalence of violence against children by parents and other close family members as well as deliberate neglect of children has been reported across the world.^1^ For example, in a United States based study, authors found primary emotional abuse in case of 26.0% of children, while emotional abuse in conjunction with child physical abuse or child neglect was found in 14.0% of cases.^9^ In another study in Minnesota, authors explored the prevalence of both incest and nonfamily abuse in two cohorts of adolescents in the 1990s. Findings indicate that sexual abuse was reported by both male and female children and among children of all ethnic groups. Approximately 10.0% of adolescents reported sexual abuse in each cohort, with girls 5 times more likely to report abuse than boys.^10^ A history of physical and/or sexual abuse was reported by 22% of male and 18% of female Amerasians. Abused male Amerasians reported significantly higher levels of psychological distress than nonabused male Amerasians, while abused and nonabused female Amerasians did not differ in their levels of psychological distress.^11^ The World Health Organization (WHO) estimates that 150 million female children and 73 million male children under 18 have experienced forced sexual intercourse or other forms of sexual violence involving physical contact, though this certainly is an underestimation.^12^ A good number of children worldwide also experience violence within the educational institutions.^13^

Intra-parental violence has a tremendous negative effecton children. For example, one study found that partner psychological abuse is strongly related to child maltreatment. Children experienced a substantially increased risk of maltreatment when partner psychological abuse was present in the homes.^14^ A United Kingdom based national survey revealed that mothers and fathers were most often responsible for physical violence, although violence by siblings was also reported.^15^ In another study it was found that the victim’s fathers were the perpetrators of sexual abuse against children in 44.0% of the cases, and the stepfather in 40.0% of the cases.^16^

Cultural beliefs and practices play an important role behind child abuse in Indian society. In a study carried out in Kolkata, it was found that 30.0% of male and 16.7% of female teachers still believe in applying physical punishment to discipline the children in school.^17^ In addition, Indian children become victim of various forms of abuse and violence. Child trafficking for commercial sexual exploitation and child labour are some of the serious problems children from poor socio-economic background experience in India. A number of researchers carried out studies on different dimensions of abuse and neglect in India. Although sample size of most of the studies was small, the findings of those studies give a fairly good idea about the nature and extent of the problem. For example, a study of 35 trafficked children and young women found that trafficking is usually conducted through offers of false marriages and jobs, or through outright abduction and sale.^18^ In another study authors found six out of the 41 trafficked children were HIV positive.^19^ A study covering a group of 120 migrant child labourers working in households, tea stalls, garages and shops in South Kolkata reveals that an overwhelming number of the children were abused either physically, psychology-cally, or sexually. Of those with health problems, a good number of them remain untreated. In some cases, the abuse and neglect was fatal.^7^ The magnitude of street children in the metropolitan cities in India is steadily increasing and they remain vulnerable to all forms of abuse, exploitation and maltreatment. Life on the streets exposes children to different exploitative and life-threatening situations.^8^ Studies on gender differences in India suggest that female children suffer relatively greater neglect than male children do throughout their early childhood. They are breast-fed less frequently than male children and for shorter duration.^20^ Interestingly, a recent study revealed that contrary to popular belief, working mothers give more quality time to their children than non-working mothers.^21^ Research has clearly indicated that a person with a major mental health problem can not act as a responsible parent. When those people become parents, they are unable to discharge their responsibilities and their children become vulnerable to neglect and violence.^22^

Very few studies have been carried out in India about nature and magnitude of violence experienced by the children in families. In Tripura, no such study has been carried out on the issue till date. Therefore, the present study attempted to understand the nature and extent of violence experienced by the children in families in Agartala, Tripura. In addition, the study tried to understand the relation of socio-economic factors with child abuse. The study also tested following two hypotheses:

1. Male and female children do differ significantly in terms of psychological, physical and sexual violence.

2. There is a significant relationship between family type, perceived family environment and parental personality, socio-economic background of the children and violence against children.

## Methods

**Study Area**

The present study was confined to eight selected secondary schools in Agartala city, Tripura. Tripura is a small state of three million people located in the Eastern part of India. The majority of religious groups in Tripura are Hindu and the state is bound by Bangladesh on three sides. Tripura is a socio-economically disadvantaged state compared to the rest of the states in India, because of its geographical location and poor transportation facilities. The literacy rate in Tripura is 73.1%, 81.0% for males and 64.9% for females, which is higherS Sthan the average Indian literacy rate i.e., 63.0% in India and 65.0% in the North East. There have been substantial improvements in school attendance rates in Tripura between 1991 and 2001. The proportion of children in the age group of 6-14 years who are not attending school halved during this decade, from 43.4% in 1991 to 23.0% in 2001. There was also a decline in the absolute number of children not attending school, from 0.254 million in 1991 to 0.164 million in 2001. There have been major increases in attendance rates in rural areas, including among female children. For example, among 6-14 year children, 62.0% of female children in the rural areas attended school in 1991; by 2001, the proportion has risen to 74.0%.^23^

**Study Sample**

A total of 320 children (160 males and 160 females), ages 14-19, had participated in the study voluntarily. Children were studying in Class eight and nine and they were selected using a multi-stage random sampling technique such that the final sample was drawn from four English and four Bengali medium high schools in Agartala, Tripura. An equal number of children were selected from each school i.e., 20 children each (10 males and 10 females) from Class VIII and IX following simple random sampling technique         

**Study Tool**

Specially designed ‘Semi-structured Questionnaire for Children/Students’ was used for collection of information for achieving the objectives of the study.^24^

The Semi-structured Questionnaire for Children/Student was originally developed in English by the authors to gather information about socio-economic and familial background of the children. In addition, the schedule was designed to gather information regarding the nature of violence experienced by the children in Tripura, at home and about reporting. The questionnaire was then translated into the local Bengali language and checked by three experienced bilingual researchers. Second, the Bengali version was back translated into English and was reviewed by two experts to confirm its equivalence with the original. This questionnaire has three broad sections like:

**Section 1:**Background Information: This section focuses on the demographic and socio-economic profile of the participants, for example, age, number of siblings, type of family, academic performance of the children, educational background of parents, occupation of parents, monthly income and living environment (rural or urban area), the child’s perception about family environment, parents’ personality and parents’ dependence behavior on substances, if any.

**Section 2:**Nature of Violence Experienced by the Child: This section consists of fifteen items and is designed to gather data from the children about the psychological, physical and sexual violence experienced by them, the nature and frequency of violence, and the profile of the perpetrators.

**Section 3:**Reporting: This part comprises of five items wherein emphasis is given on the reporting of the incident to suitable authorities like law enforcement officials or local non-governmental organizations, and the reasons for not reporting the incident to them.

The choice for giving responses in the questionnaire is of two broad categories like dichotomous response and multiple choice responses. Some of the variables are followed by three to six alternative responses. The subject is instructed to put a tick mark against the answer, which is best suited for him/her. In case of dichotomous response, the subject gets a score of 1 if the answer is ‘yes’ and a score of 2 for answering ‘no’. For example, in case of age, subject is asked to put a tick mark against appropriate category of response (1 if belongs to ‘14-16 years’; 2 if belongs to ‘16-17 years’ and 3 if belongs to ‘18 years and above’) while in case of type of family, subject is supposed to give a tick mark against the sui category of response; 1 if the answer is ‘joint family’ and a score of 2 for answering ‘nuclear family’. Academic performance of the children is measured in terms of two point scale ranging from 1 - “good” (60% and above marks in the last examination); and 2 – “no so good” (below 60% marks in the last examination). In case of parent’s personality as perceived by the children, dichotomous response is used against two broad categories of personality like (i) dominating, short-tempered, and/or aggressive and (ii) friendly and/or submissive.

**Operational Definition**

** Physical Violence:**In the present study physical violence means any parent or caregiver act causing non-accidental physical injury which includes pushing, grabbing, kicking, hitting, choking, locking, burning and punishing the child in any painful way.

**Psychological Violence:**Emotional/psychological violence means the habitual verbal harassment of a child by disparagement, criticism, threat and ridicule. In the present study, emotional or psychological violence includes behavior that threatens or intimidates a child such as threats, name calling, belittling, teasing, and shaming.

**Sexual Violence:**Sexual violence refers to sexual exposure of contact by a person older than a child for the purpose of sexual stimulation or exploitation regardless of the use of force or any accompanying physical injury. In the present study, sexual abuse of children has been defined as touching of private parts, fondling, penetration and rape.

**Procedure**

First, permission from the authorities of different institutions was sought for data collection, and then a tentative time schedule was developed for data collection. Data were collected from the children during February to June 2008 following the self-administration method after obtaining their consent. It took about 15-20 minutes times for the children to fill up the questionnaire. The ethical issues which were taken into account ensuring quality data included briefing the participants about study objectives, obtaining informed consent, ensuring confidentiality and explaining that participants were free to withdraw at any point during the study period, if they wished. Data were collected by the authors and in case of any query authors clarified the issue. In the questionnaire there was no column for writing name and address of the children.

**Data Analysis**

The data collected from the children were subjected to an in-house thorough review by the researchers for checking whether the children answered all the questions. None of the questionnaire was found incomplete. Thereafter, data were entered with accuracy and precision into the computer for computation. Chi-square test was used for data analysis using SYSTAT package and for testing of hypotheses.

## Results

**Background Information**

Most of the children (92.5%) belonged to the 14-15 year age group while the rest, 5.9% and 1.6%, belonged to 16-17 years and 18 years and above age groups. Regarding family size, almost an equal number of the children came from joint (46.2%) and nuclear/single families (53.8%). Nuclear family means where husband and wife live only with their children, while a joint family is a set up of husband and wife along with their children and extended family consisting of two or more generations and food is cooked for all of the members in one kitchen.

So far as the academic performance of the children in the last examination is concerned, it has been observed that the performance of 19.5% of the children had been very good i.e., they had got, on an average, 70.0% marks and above. The performance of the 41.9%, 24.4%, and 14.4% of the children had been good, moderate and poor, respectively.

With regards to parents’ education, approximately 50.0% of the fathers and 35.3% of the mothers of the respondents were graduates (that is, they successfully completed college education). About 37.5% of the fathers and 27.9% of the mothers had completed postgraduate education, while rest of the parents had not completed graduate level education. Regarding the occupation of the parents, data indicate that 70.0% of the respondents’ fathers and 40.6% of the mothers were in the service industry, whilst about 25.3% of the fathers and 10.3% of the mothers were engaged in business. More than one-fourth of the mothers (28.8%) were housewives. The rest of the parents were either engaged in casual work or were unemployed. The total monthly family income for the majority of the children ranged from Rs.10,001/ to 20,000/ per month (US$ 200 to 400) while the monthly family income for 5.0% of the children’s family was Rs.20,001/ per month and above (above US$ 400). The monthly family income for about 26.0% of the respondent’s family was below Rs.10,000 (below US$ 200). The majority of the families lived in urban (51.1%) and semi-urban areas (21.1%).

Since any form of violence against children is directly or indirectly related to family environment and parental personality, in the present study the same issues were explored. Interestingly, findings revealed that 38.7% of the children reported that they perceived their fathers’ personality to be very dominating, short-tempered and/or aggressive. In contrast, the children’s perception about mother’s personality was not so dominating. Only 15.7% of the children found their mother’s personality to be dominating, short-tempered and/or aggressive. Although the family environment was reportedly found to be congenial by the majority of the children (91.6%), 8.4% reportedly experienced disturbances and/or tension in the family.

**Information Pertaining to Violence against Children**

The present study made an attempt to understand the prevalence of psychological, physical and sexual violence experienced by the children in Tripura. Regarding psychological violence, about 20.9% (67/320) of the children (21.9% males and 20.05 females) reported being victims of these types of violence in the family (Table 1). Out of a total of 67 psychologically abused children, 37.3% experienced the same almost regularly, while the rest 41.8% and 20.9%, experienced the same occasionally and rarely respectively. In this regard, not much gender-related difference was found (p-value: 0.6802) (Table 1).

**Table:** Table 1: **Psychological violence as reported by the children (N=320)**

Psychological Violence	Male Child (N=160)		Female Child (N=160)		Total (N=320)	
	f.	%	f.	%	f.	%
Yes	35	21.9	32	20.0	67	20.9
No	125	78.1	128	80.0	253	79.1
If yes, how frequently?	(N=35)		(N=32)		(N=67)	
Almost regularly (i.e., once or twice a week)	13	37.1	12	37.5	25	37.3
Occasionally (i.e., once a month)	15	42.9	13	40.6	28	41.8
Rarely (i.e., once in six months)	7	20.0	7	21.9	14	20.9
If yes, what happened? For instance: Somebody known or unknown to you (MRP*)	(N=35)		(N=32)		(N=67)	
called your names, said mean things or cursed	15	31.3	13	34.2	28	41.8
made you feel ashamed in front of other people	18	37.5	14	36.8	32	47.8
said they wished you were dead	6	12.5	-	-	6	9.0
threatened to leave or abandon you	5	10.4	5	13.2	10	14.9
threatened to hurt or kill you	1	2.1	-	-	1	1.5
bullied or teased you	3	6.3	6	15.8	9	13.4
Other forms, please specify	-	-	-	-	1	1.5
If yes, who did the same with you?	(N=35)		(N=32)		(N=67)	
Father	12	25.0	8	21.1	20	29.9
Mother	11	22.9	15	39.5	26	38.8
Elder Sibling	2	4.2	4	10.5	6	9.0
Teacher	14	29.2	8	21.1	22	32.8
Relatives	7	14.6	2	5.3	9	13.4
Others i.e., friends and peer group members	2	4.2	1	2.6	3	4.5

*Multiple Responses Possible

As far as the nature of psychological violence is concerned, it was mostly in the forms of verbal abuse included calling names, cursing and humiliating the child in front of others, threatening to abandon, bullying and teasing. Psychological violence was mostly perpetrated by the parents and the teachers, followed by relatives and elder siblings. Gender-wise analysis of data (Table 1) revealed that male children faced violence mostly from the teachers (29.2%), followed by the parents (25.0% by fathers and 22.9% by mothers) while the female children were abused mostly by mothers (39.5%), followed by the teachers (21.1%) and elder siblings (10.5%).

As far as physical violence against children is concerned, nearly 22% of the children reported that they were abused physically. Male children (24.4%) were more likely to be victims of physical abuse than their female counterparts (19.4%). Regarding the frequency of physical violence, about one-fourth (25.7%) of the children reported that they were abused regularly, while about half (47.1%) experienced physical violence occasionally; the rest 27.1% experienced it rarely (Table 2).

**Table:** Table 2: **Physical violence as reported by the children (N=320)**

Psychological Violence	Male Child (N=160)		Female Child (N=160)		Total (N=320)	
	f.	%	f.	%	f.	%
Yes	39	24.4	31	19.4	70	21.9
No	121	75.62	129	80.62	250	78.1
If yes, how frequently?	(N=39)		(N=31)		(N=70)	
Almost regularly (i.e., once or twice a week)	10	25.6	8	25.8	18	25.7
Occasionally (i.e., once a month)	19	48.8	14	45.2	33	47.1
Rarely (i.e., once in six months)	10	25.6	9	29.0	19	27.1
If yes, what happened? For instance: Someone known to you has (MRP*)	(N=39)		(N=31)		(N=70)	
pushed, grabbed or kicked you	5	12.8	17	54.8	22	31.4
hit or beaten you, especially with a belt, stick or other object	15	38.5	4	12.9	19	27.1
choked you or tried to strangle you	-	-	-	-	-	-
locked, tied or chained you to something	2	5.1	-	-	2	2.9
burnt or bruised you with a hot or sharp object	-	-	-	-	-	-
punished you in any other painful way	18	46.2	13	41.9	31	44.4
other forms	4	10.3	7	22.6	11	15.7
If yes, who did the same with you? MRP*	(N=39)		(N=31)		(N=70)	
Father	14	35.9	6	19.4	20	28.6
Mother	5	12.8	7	22.6	12	17.1
Elder Sibling	2	5.1	6	19.4	8	11.4
Teacher	18	46.2	12	38.7	30	42.9
Relatives	2	5.1	4	12.9	6	8.6
Others i.e., friends and peer group members	1	2.6	8	25.8	9	12.9

*Multiple Responses Possible

Regarding the form of physical violence, over 46% of the male children reported that they were subject to painful punishments such as being beaten with a belt, stick or other objects (38.5%) while female children were mostly pushed (46.2%), grabbed (41.9%) or kicked (54.8%). As for the perpetrators of violence are concerned, male children were physically abused mostly by the teachers (46.2%), followed by fathers (35.9%) and mothers (12.8%). In contrast, female children were abused mostly by the teachers (38.7%), followed by others (25.8%), mothers (22.6%), fathers (19.4%) and elder siblings (19.4%).

About one-fourth of the female children (25.0%) and 11.3% of the male children were reported to be victims of sexual violence. About 20.0% of the sexual violence experienced by the female children occurred regularly, while 32.5% and 47.5% were abused occasionally and rarely, respectively. On the other hand, 33.3% of the male children reported being sexually abused “once in a month”, where as 66.7% reported such abuse occurring approximately “once every six month” (Table 3).

**Table:** Table 3: **Sexual Violence as Reported by the Children (N=320)**

Sexual Violence	Male Child (N=160)		Female Child (N=160)		Total (N=320)	
	f.	%	f.	%	f.	%
**Yes**	18	11.3	40	25.0	58	18.1
No	142	88.75	120	75.00	262	81.9
If yes, how frequently?	(N=18)		(N=40)		(N=58)	
Almost regularly (i.e., once or twice a week)	-	-	8	20.0	8	13.8
Occasionally (i.e., once a month)	6	33.3	13	32.5	19	32.8
Rarely (i.e., once in six months)	12	66.7	19	47.5	31	53.4
If yes, what happened? For instance: Someone known or unknown to you (MRP*)	(N=18)		(N=40)		(N=58)	
touched or looked at you private parts	6	33.3	9	22.5	15	25.9
asked or forced you to touch or look at the private parts	8	44.4	13	32.5	21	36.2
made you feel bad/ uncomfortable by speaking or showing you adult picture	7	39.0	14	35.0	21	36.2
forced you to have sexual intercourse	-		8	20.0	8	13.8
other forms of abuse	-		-	-	-	-
If yes, who did the same with you? MRP*	(N=18)		(N=40)		(N=58)	
Father	-	-	-	-	-	-
Mother	-	-	-	-	-	-
Elder Sibling/cousin	6	33.3	-	-	6	10.3
Teacher	3	16.7	15	37.5	18	31.0
Relatives	7	38.9	22	55.0	27	46.6
Others (Friends and peer group members)	2	11.1	3	7.5	5	8.6

*Multiple Responses Possible

There were some similarities between the male and the female children in terms of nature of sexual abuse. In case of both male and female children, somebody touched or looked at their private body parts, or asked them or forced them to touch their private body parts. More than one-thirds of the children, irrespective of gender, were shown adult pictures, which made them feel bad and/or uncomfortable. About 20.0% of the female children were forced to have sexual intercourse with the perpetrator.

The male children were sexually abused mostly by relatives (38.9%), elder siblings/cousins (33.3%), and teachers (16.7%) while the female children were sexually abused mostly by the relatives (55.0%), followed by the teachers (37.5%).

Data pertaining to psychological, physical and sexual violence experienced by the children have been depicted in Table 4 for testing of the hypothesis (that is, ‘male and female children do differ significantly in terms of psychological, physical and sexual violence’).

**Table:** Table 4: **Nature of violence experienced by the children**

Nature of Violence	Sample Group		Total (n=320) %	x2 (p-value)	Level of Significance
	Male (n=160) %	Female (n=160) %			
Psychological	21.9 (35/160)	20.0 (32/160)	20.9 (67/320)	0.17 (0.6802)	NS
Physical	24.4 (39/160)	19.4 (31/160)	21.9 (70/320)	1.17 (0.2793)	NS
Sexual	11.3 (18/160)	25.0 (40/160)	18.1 (58/320)	10.19 (0.0014)	0.01

Significant at 0.01 level: NS: Not significant

Using chi-square tests, statistically no significant association between psychological (x^2^=0.17; p-value: 0.6802) and physical (x^2^ =1.17; p-value: 0.2793) violence across gender was found. However, statistically significant association was found between being female and experiencing sexual violence (x^2^=10.19; p-value: 0.001).

**Witness of Family Violence**

About 29.7% of the children reported that they witnessed violence in the family mostly between the parents. In this regard, male children witnessed more violence (35.0%) compared to female children (24.4%). About 8.4% of the children, irrespective of gender, witnessed violence regularly, (that is, once or twice a week), while 34.7% witnessed it occasionally (that is, “once a month” or “once in six months”). So far as manifestation of the violence is concerned, children mostly reported that the parents shouted at each other, followed by beating, kicking, or other forms of physical abuse.

**Reporting of Violence**

Out of a total of 58 sexual abuse cases as disclosed by the children, only 9 cases (15.5%) were reported to the police or school authorities by the parents. The other 49 cases were not reported to the police or to the school authorities, owing to a number of factors such as fear of social stigma (52.9%), perceived harassment (35.3%), parents’ unwillingness (35.3%), parents’ disbelief about cooperation from the police (23.5%), and fear of threats from the perpetrator (15.7%). Only 2 cases out of 58 were reported to the local non-government organizations (NGOs). The reasons for poor reporting of the cases of sexual abuse to the NGOs include social stigma and perceived harassment. About one-third of the cases clearly stated that non-availability of NGO’s in the locality was the main reason for poor reporting of the incidents of sexual abuse. At the same time, 14.3% of the children clearly stated that their parents did not report the incident to the NGOs out of a feeling that the people at the NGO would not believe them. So far as the outcome of reporting of the incident to the police or school authorities is concerned, out of nine reported cases, official steps were taken only in three cases, and only in one case was the perpetrator arrested. As for the other five cases, the school authorities warned the respective school teacher, and in case of private teachers, parents were advised to dismiss the private teacher.

**Relation of Violence with Socio-economic Factors**

Comparative analysis of the data using chi-square tests with regard to family type, monthly income, perceived family environment and academic performance of the children who experienced physical violence and who did not experience the same, disclosed some interesting features. Children were more likely to experience physical and sexual violence in the single/nuclear families with disturbed family environment (p<0.01).

Regarding the monthly income of the families, data show that the children from high-income families experienced more physical violence (p<0.01) while children from the lower income group of families experienced more psychological violence (p<0.01). Psychological violence and corporal punishment have been regarded as normal cultural practices in some families in Tripura. Comparative analysis of data with regard to sexual violence indicated that children across all income groups were equally vulnerable to sexual violence (p>0.01) (Table 5,6,7).

**Table:** Table 5: **Relationship of physical violence against children with socio-economic and other factors**

Variables	% of Abused	RR*	x2	p-Value
(A) Family Type:				
Joint Family	15.54% (23/148)	1.00	6.46	0.0110
Single Family	27.33% (47/172)	1.76		
(B) Monthly Income:				
Up to Rupees (Rs.) 10,000/	12.20% (10/82)	1.00	15.13	0.0005
Rs.10,001/- to Rs.20, 000/	22.83% (50/219)	1.87		
Rs.20,001/- & above	52.63% (10/19)	4.32		
(C) Perceived Family Environment:				
Congenial	16.72% (49/293)	1.00	53.92	<0.0001
Disturbed	77.78% (21/27)	4.65		
(D) Perception about Mother’s Personality:				
Friendly and submissive	11.11% (30/270)	1.00	117.15	<0.0001
Dominating, short-tempered and aggressive	80.00% (40/50)	7.20		
(E) Perception about Father’s Personality:				
Friendly and submissive	6.12% (12/196)	1.00	73.44	<0.0001
Dominating, short-tempered and aggressive>	46.77% (58/124)	7.64		
(F) Academic Performance:				
Good	13.78% (27/196)	1.00	19.42	<0.0001
Not so good	34.68% (43/124)	2.52		

*RR: Relative Risk

**Table:** Table 6: **Relationship of Psychological Violence against Children with Socio-economic and Other Factors**

Variables	% of Abused	RR	x2	p-Value
A) Family Type:				
Joint Family	15.54% (23/148)	1.00	1.00	0.3185
Single Family	27.33% (47/172)	1.26		
(B) Monthly Income:				
Up to Rs. 10,000/	50.00% (9/18)	1.00	9.82	0.0074
Rs. 10,001/-to Rs.20, 000/	19.52% (49/251)	0.39		
Rs. 20,001/- & above	17.65% (9/51)	0.35		
(C) Perceived Family Environment:				
Congenial	18.84% (55/292)	1.00	8.91	0.0028
Disturbed	42.86% (12/28)	2.28		
D) Perception about Mother’s Personality:				
Friendly and submissive	3.21% (7/218)	1.00	129.82	<0.0001
Dominating, short-tempered and aggressive	58.82% (60/102)	18.32		
(E) Perception about Father’s Personality:				
Friendly and submissive	7.59% (11/145)	1.00	28.55	<0.0001
Dominating, short-tempered and aggressive	32.00% (56/175)	4.22		
(F) Academic Performance:				
Good	10.63% (27/254)	1.00	79.04	<0.0001
Not so good	60.61% (40/66)	5.70		

**Table:** Table 7: **Relationship of sexual violence against children with socio-economic and other factors**

Variables	% of Abused	RR	x2	p-Value
(A) Family Type:				
Joint Family	15.54% (23/148)	1.00	8.18	0.0042
Single Family	27.33% (47/172)	2.08		
(B) Monthly Income:				
Up to Rs. 10,000/	15.85% (13/82)	1.00	1.15	0.5636
Rs. 10,001/- to Rs.20, 000/	18.26% (40/219)	1.15		
Rs. 20,001/- & above	26.32% (5/19)	1.66		
(C) Perceived Family Environment:				
Congenial	16.04% (47/293)	1.00	10.16	0.0014
Disturbed	40.74% (11/27)	2.54		
(D) Perception about Mother’s Personality:				
Friendly and submissive	7.41% (20/270)	1.00	133.75	<0.0001
Dominating, short-tempered and aggressive	76.00% (38/50)	10.26		
(E) Perception about Father’s Personality:				
Friendly and submissive	8.16% (16/196)	1.00	33.82	<0.0001
Dominating, short-tempered and aggressive	33.87% (42/124)	4.15		
(F) Academic Performance:				
Good	10.71% (21/196)	1.00	18.72	<0.0001
Not so good	29.84% (37/124)	2.78		

The academic performance of the children was not as good as compared to that of non-abused children (p<0.01), irrespective of nature of violence. Therefore, it might be stated that violence against children could be one of the reasons for poor academic performance of the abused children.

The parent’s personality was found to have a direct association with all types of violence against children (p<0.01). Children who experienced violence, perceived their parents’ personality, both that of the father and the mother, to be dominating, short-tempered and aggressive – a trend not found in the children who did not experience violence at home.

Therefore, it may be stated that the second hypothesis is retained (that is, there is a significant relationship between family type, perceived family environment, perceived parental personality, socio-economic background of the children and violence against children).

## Discussion

About one-fifth of the children in Tripura experienced psychological (20.9%), physical (21.9%) or sexual (18.1%) violence. In a national level study in India on violence against children, it was found that two out of every three children and one out of every two children experienced physical and psychological violence. In case of incidents of sexual abuse, it was one in two children.^25^ Compared to national level data in India, the incidence rates of physical, psychological and sexual in Tripura were much lower. One of the reasons behind psychological violence against children could be the influence of cultural practice, the dominating role of older people in Indian society, over expectations of parents pertaining to academic performance, and an undermining of the status of children. In general, children across different social strata in India are treated as incapable of knowing what is best for them, and therefore there is a need to control their behavior and decisions in every aspect of their life. This mainly occurs in middle and upper social class families, while children from lower social strata generally have parents who remain indifferent about the welfare of the children especially in the case of female children. In the lower social strata, female children are discriminated in terms of nutrition, education and health care.^26,27^ Regarding the type of psychological violence, calling names, saying mean things, cursing and humiliating the children in front of others were the most prevalent ones.

Parents and teachers most commonly abused children psychologically, followed by relatives and elder siblings. Some of the words used by the private teachers or parents in disciplining a child are acceptable in the local culture, but by the latest definition of these words,^28,29^ they are termed as psychological violence against children. Although male children were reported as being more vulnerable to psychological violence, statistically no significant difference was found between male and female children in terms of psychological violence. This issue requires proper attention of the school authorities, to change the cultural practice, which has yet to be recognized as abusive.

Socio-economic data clearly indicate that most of the children from high income group of families are 4.32 times more vulnerable to physical violence compared to children from low income group of families. In the high income group of families, parents have high expectations of their children in regard to their academic performance, for which sometimes they use physical/corporal punishment. The findings of the present study with regard to incidence of physical violence are almost similar with the findings of another US-based study.^11^ However, incidence of physical violence in some other countries is found to be much higher. For example, in a survey of students aged 11 to 18 years in the Kurdistan Province of the Islamic Republic of Iran, 38.5% of the subjects reported experiences of physical violence at home that had caused mild to severe physical injury.^30^ With respect to forms of physical violence, gender-wise differences have been observed; as male children were mostly beaten with belts, sticks or other objects, and female children were mostly pushed (46.2%), grabbed (41.9%) and kicked (54.8%). A review of the research on physical victimization of children in the Republic of Korea found that kicking, biting, choking and beating of children by parents are alarmingly common, of which a small proportion results in disability.^31^

Some of the previous studies in Kolkata, India revealed an association between socio-economic background and child maltreatment in the form of trafficking. Children from poor families with low level of education and poor social network are more prone to child trafficking, abuse and maltreatment as they can be easily allured in the name of marriage and job offers in the urban areas.^16,32^

Apart from teachers, fathers were found to be more abusive towards male children in Tripura, while in case of female children it was the mother. Findings of a national survey in the UK found that mothers and fathers were most often responsible for physical violence, although violence by siblings was also reported.^15^ There are also some similarities in the findings of the present study and that of another study with respect to the profile of the perpetrators and the nature of sexual abuse of female children.^16^ A review of epidemiological surveys from 21 countries, mainly high and middle-income countries, found that at least 7.0% of females (ranging up to 36.0%) and 3.0% of males (ranging up to 29.0%) reported sexual victimization during adulthood. According to these studies, between 14.0% and 56.0% of the sexual abuse of female children, and up to 25.0% of the sexual abuse of male children, was perpetrated by relatives or step-parents.^34^ The enormity of the problem of child sexual abuse has been described as one of the most discouraging discoveries of our era.^35^ A number of studies clearly indicted the short-term and long-term impact of abuse on mental health of the children.^19,36-39^

It is also clear from the findings of previous national and international studies that corporal punishment is a predictor of depression, unhappiness, anxiety and feelings of hopelessness in children and youth.^16,40,41^

In a Hong Kong study author found that the rates of parent-to-child physical aggression were 57.5% for corporal punishment and 4.5% for physical maltreatment. Mothers, as compared to fathers, reported higher rates and more frequent use of corporal punishment on their children.^42^ it could be because of high stress of mothers in daily life. In the middle class families in Tripura, the majority of the mothers are working outside for earning money to run the family comfortably after doing all household chores and looking after the children. As a result, sometimes some of the mothers lose their patience easily and abuse their children for committing minor mistakes and / or for better academic performance.

Both male and female children were found to be victims of sexual violence in Agartala, Tripura although female children were more frequent to be sexually abused (20.0%) than male children. In general, in Tripura, there is a common practice to appoint three to four private teachers for the children for better academic performance. Children either go to the teacher’s home or teachers come to the home of the children for special guidance, and in most of the cases, teachers teach the children in a separate room to avoid any disturbance. This sort of privacy, however, provides an opportunity for sexual violence against children in the hand of private teachers.

There are some similarities between the nature of sexual violence against male and female children, except for forced sexual intercourse, which has been experienced by one-fifth of the female children. In the case of both male and female children, somebody touched or looked at their private body parts, or asked or forced them to touch their private body parts. More than one-third of the children, irrespective of gender, were shown adult pictures, which made them feel bad and/or uncomfortable. The WHO estimates that 150 million female children and 73 million male children under 18 have experienced forced sexual intercourse or other forms of sexual violence involving physical contact, though this is certainly an underestimation. The family members or other people residing in or visiting a child’s home - people normally trusted by the children and often responsible for their care - are responsible for much of this sexual violence.^12^

The study also disclosed some association between violence against children and family environment, type of family, parent’s personality and educational performance of the children in Agartala, Tripura. Although no such clear trend has been observed regarding the monthly income of the families, data has revealed that children from the high-income families experienced more physical violence (p<0.01) while children from low income families experienced mostly psychological violence (p<0.01). So far as perceived family environment is concerned, a comparative picture clearly indicates that uncongenial and/or disturbed family environment has some positive correlation with violence against children, irrespective of the nature of the violence. The academic performance of the children who experienced any forms of violence was poorer in case of abused children as compared to that of children who did not experience the same (p<0.01). In this regard, authors of another study observed that a substantiated maltreatment report is significantly associated with poorer academic performance (p<0.01) and poorer adaptive functioning (p<0.01).^43^

Children who experienced violence perceived their parents’ personality, both father’s and mother’s, to be dominating, short-tempered and/or aggressive, unlike the children who did not experience the same. Findings of the present study with regard to parental personality and family environment are consistent with the findings of some of the previous studies.^1,13^ Parents with poor impulse control, low self-esteem and mental health problems are more likely to use physical violence against children.^43^ Parents who use violence against their children may well have experienced violence as children themselves.^44^

More than one-forth (29.7%) of the children reported that they witnessed violence in the family mostly between the parents. There is increasing concern about the impact of marital conflict on children. A number of studies reported adjustment problems, aggression, social withdrawal and feeling of insecurity among the children who witnessed violence in the family.^45,46^

Reporting of sexual abuse (15.5%) was very low mostly because of social stigma, perceived harassment, unwillingness of parents, disbelief of parents and threat by perpetrators. Reporting of cases of sexual abuse is low worldwide.^16^ In India in general, and in Tripura in particular, most of the police stations do not have a private area to conduct interviews with the child, and the lack of female police officials is a huge impediment in investigating and recording incidents of child abuse.

## Conclusions

About one-fifth of the children experienced psychological, physical or sexual violence or a combination of violence in Agartala, Tripura. Male children were more often victims of psychological and physical violence while female children experienced more sexual violence. Male and female children differed significantly in the case of sexual violence only (p<0.01). Data clearly indicated relation of violence against children with single/nuclear families, uncongenial and/or disturbed family environment and dominating, short-tempered and/or aggressive parents’ personality, irrespective of nature of violence. Children from high income group of families experienced more physical violence while children from the middle income group of families experienced more psychological violence. Sexual violence was found equally prevalent in all socio-economic groups. About one-third (29.7%) of the children reported witnessing violence in the family. The academic performance of the violence- experienced children, irrespective of the nature of violence and socio-economic groups, was found to be poorer compared to academic performance of non-violence-experienced children. The findings of the study suggest that institutional authorities in Tripura should take up culturally sensitive intervention program for creating awareness among parents and teachers about the issue. The government should enact a law for mandatory reporting of child maltreatment by the teachers, doctors and nurses.

**Limitations**

There are some limitations of the present study that should be noted. First, the sample size of the present study was small for a school-based study. Second, the sample comprised of school attending children from urban areas only, therefore, the diversity of the sample was limited in terms of socio-economic class, ethnic background and educational status. Thus, the findings may not be generalizable to all the school children in the community. Third, the findings of the study were based on the “self reports” of the participants. Although the issue of confidentiality was strictly followed, some children may not reveal the incidents of violence they experienced especially sexual violence because of their high emotional attachment with either of the parents and/or feeling of embarrassment. Another study with large sample covering children from both rural and urban areas across ethnic background and educational status is suggested to have a better understanding of the issue.
